# Rapid Identification of Superior Endogenous Signal Peptides for Heterologous Protein Secretion by *Corynebacterium glutamicum* Through Modular Cloning and Automation

**DOI:** 10.1111/1751-7915.70299

**Published:** 2026-01-16

**Authors:** Susana Matamouros, Julia Tenhaef, Astrid Bida, Stephan Noack, Michael Bott

**Affiliations:** ^1^ Institute of Bio‐ and Geosciences, IBG‐1: Biotechnology, Forschungszentrum Jülich Jülich Germany; ^2^ The Bioeconomy Science Center (BioSC), Forschungszentrum Jülich Jülich Germany

**Keywords:** automation, *Corynebacterium glutamicum*, modular cloning, PETase, recombinant protein secretion, signal peptide library

## Abstract

Secretory protein production by microbial hosts simplifies product recovery and is therefore preferred over intracellular production. Efficient secretion of heterologous proteins by bacteria requires the identification of optimal signal peptides (SPs), a step that often limits process development. Using *Corynebacterium glutamicum* as a model host, we established a modular cloning system enabling rapid assembly of expression plasmids for secretory protein production. Screening a library of 30 individually cloned endogenous SPs with a fungal cutinase as target protein demonstrated that several native SPs achieved substantially higher secretion levels than the widely used *Bacillus subtilis* NprE reference SP. To accelerate SP discovery, we developed a one‐pot approach in which *C. glutamicum* was directly transformed with a single modular cloning mixture containing all 30 SPs. Combined with the AutoBioTech high‐throughput platform for cultivation, harvesting, and protein quantification, this strategy enabled screening of several hundred clones in parallel. Superior SPs were rapidly identified not only for cutinase but also for four polyethylene terephthalate hydrolases (PETases). This streamlined workflow significantly reduces time and cost for selecting effective SPs and provides a versatile platform for advancing secretory protein production in *C. glutamicum* .

## Graphical abstract

A modular cloning approach combined with an automated high‐throughput platform enabled rapid identification of superior endogenous signal peptides for the secretion of proteins of interest (POIs), such as industrial enzymes, by *Corynebacterium glutamicum*. This streamlined workflow accelerates discovery and development of efficient bacterial systems for secretory protein production.

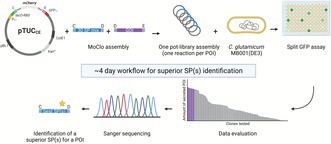

## 1 Introduction

*Corynebacterium glutamicum* , a non‐pathogenic soil actinobacterium, is used since the 1960s for the production of amino acids, in particular L‐glutamate and L‐lysine (Eggeling and Bott 2015; Wendisch et al. 2016; Becker et al. 2018). Owing to its industrial relevance and proven robustness in large‐scale fermentation, *C. glutamicum* has become an intensively studied model organism in biotechnology (Eggeling and Bott 2005; Burkovski 2008; Yukawa and Inui 2013) and strains have been developed for the synthesis of a wide range of chemicals (Wieschalka et al. 2013; Becker et al. 2018; Wolf et al. 2021). Beyond small molecule production, *C. glutamicum* has also gained increasing attention as host for recombinant protein production and secretion (reviewed by Liu et al. 2016; Freudl 2017; Lee and Kim 2018; Lee and Jeong 2023).

*C. glutamicum* harbours both the general secretion or Sec pathway and the twin‐arginine translocation or Tat pathway, for the transport of proteins across the cytoplasmic membrane (Freudl 2017). Secretion of heterologous proteins into the culture supernatant has several advantages, most notably the simplification and significant cost reduction of downstream processing for product recovery (Freudl 2018). In this context, cell disruption is unnecessary, continuous protein production is possible, and dilution in the medium mitigates the risk of protein aggregation. Particularly in the food and biopharmaceutical sectors, *C. glutamicum* provides important benefits over hosts such as *Escherichia coli* or *Bacillus subtilis* , since it neither produces endotoxins nor exhibits significant extracellular protease activity, respectively (Suzuki et al. 2009). Reflecting this potential, the Japanese company Ajinomoto Co. Inc. employs *C. glutamicum* in its proprietary “CORYNEX” system for commercial protein production.

A crucial step in developing secretory production processes for heterologous proteins is the identification of an appropriate SP that directs the protein to either the Sec or the Tat translocation systems in the cytoplasmic membrane (Freudl 2018). Numerous studies have demonstrated that no universally efficient SP exists for all proteins; rather, the optimal SP must be determined individually for each target protein through systematic screening (Brockmeier et al. 2006; Mathiesen et al. 2009; Degering et al. 2010; Zhang et al. 2016). Moreover, the best performing SP for a given protein can vary depending on the bacterial host. For example, in a study of ~150 SPs of *B. subtilis* tested for cutinase secretion in *C. glutamicum* , secretion efficiencies of specific SP‐cutinase combinations differed substantially between the two species (Hemmerich et al. 2016). Similarly, a library of 405 predicted SPs from *C. glutamicum* strain R was screened for secretion of α‐amylase from *Geobacillus stearothermophilus* , identifying 108 functional SPs (Watanabe et al. 2009). Remarkably, 11 of these enabled 50‐ to 150‐fold higher α‐amylase secretion compared to PS2 SP, one of the most studied SPs for heterologous protein production in *C. glutamicum* (Kikuchi et al. 2003). These findings clearly underscore that SP screening is a powerful and efficient strategy for enhancing secretion of heterologous proteins.

To streamline SP screening in *C. glutamicum* and harness its native repertoire, we established a modular cloning (MoClo) strategy for the rapid identification of optimal SPs for chosen target proteins using the recently established AutoBioTech platform (Rosch et al. 2024). MoClo is a standardised DNA assembly framework based on Golden Gate cloning, which enables the hierarchical combination of reusable genetic parts with predefined overhangs into multi‐gene constructs. The modular nature of the system, incorporating elements such as promoters, ribosome binding sites, SPs, and target proteins, facilitates both systematic evaluation and random screening of specific modules of interest.

We constructed a MoClo‐compatible SP library for the type strain *C. glutamicum* ATCC 13032 together with transcriptional unit vectors designed for direct secretion screening in *C. glutamicum* . Cutinase was employed as the primary model protein as it has been extensively used to study protein secretion in this organism (Hemmerich et al. 2016, 2019; Bakkes et al. 2021). Secreted protein was detected by two complementary methods: measurement of cutinase enzymatic activity and a split GFP assay, in which the target protein is fused at its C‐terminus to the GFP_11_ reporter tag (Cabantous et al. 2005; Knapp et al. 2017) and a fluorescent signal emitted upon incubation with the GFP_1‐10_ detector protein. Using this integrated approach, we rapidly identified highly effective SPs not only for cutinase but also for the secretion of four polyethylene terephthalate hydrolases (PETases).

## 2 Materials and Methods

### 2.1 Strains and Cultivation Conditions

*E. coli* DH5α was used for cloning procedures and cultivated in LB medium. Whenever necessary, the medium was supplemented with ampicillin (100 μg/mL) or kanamycin (25 μg/mL), 100 μM isopropyl‐β‐D‐thiogalactoside (IPTG) and 50 μg/mL 5‐bromo‐4‐chloro‐3‐indolyl β‐D‐galactopyranoside. *C. glutamicum* MB001(DE3) (Kortmann et al. 2015), a prophage‐free strain derivative of the ATCC 13032 type strain that allows for IPTG‐inducible T7‐RNA polymerase‐dependent gene expression, was used for all experiments. *C. glutamicum* was cultivated in brain heart infusion (BHI) medium (Bacto, BD, Heidelberg, Germany) or CGXII mineral medium containing per litre of distilled water: 1 g K_2_HPO_4_, 1 g KH_2_PO_4_, 20 g (NH_4_)_2_SO_4_, 5 g urea, 42 g 3‐(*N*‐morpholino)propanesulfonic acid (MOPS), 0.25 g MgSO_4_·7H_2_O, 10 mg CaCl_2_, 0.2 mg biotin, 10 mg FeSO_4_·7H_2_O, 10 mg MnSO_4_·H_2_O, 1 mg ZnSO_4_·7H_2_O, 0.3 mg CuSO_4_, 0.02 mg NiCl_2_·6H_2_O, 30 mg of 3,4‐dihydroxybenzoate (iron chelator), and 20 g/L glucose as carbon and energy source. The pH was adjusted to 7.0 with NaOH. Whenever necessary, kanamycin (25 μg/mL) and 250 μM IPTG were added.

*C. glutamicum* cultures were always cultivated at 30°C and followed three steps: seed culture, preculture, and main culture. In the seed culture, single clones were grown for 4 h in BHI supplemented with 91 g/L sorbitol (BHIS) and kanamycin, and then diluted 1:20 in CGXII with 20 g/L glucose and kanamycin for the overnight precultures, which were grown for ~16 h. Finally, the main cultures were inoculated from the overnight precultures diluted 1:20 in the same medium. Protein production was induced after 4 h cultivation by the addition of 250 μM IPTG and cultivation was allowed to proceed for an additional period of 20 h in the same cultivation conditions. The supernatant was collected by centrifugation for 5 min at 3000 *g* and stored at −20°C until usage. Cultivations were performed either in 50 mL in 500 mL baffled flasks with 130 rpm agitation or in a BioLector cultivation system (Beckman Coulter GmbH, Aachen, Germany) using 800 μL culture volume in a 48‐well FlowerPlate (Beckman Coulter GmbH, Aachen, Germany) shaken at 1200 rpm. In the AutoBioTech platform seed and precultures were cultivated in a total volume of 200 μL in 96. V‐bottom well plates (max. well volume 400 μL, Nunc, ThermoFisher Scientific, Roskilde, Denmark) and the main cultures were grown in 800 μL culture volume in 96‐square deep well plates (max. well volume 2 mL, VWR Collection, VWR International, Leuven, Belgium). Here the liquid handling was performed by a Fluent 1080 (Tecan Group Ltd., Männedorf, Switzerland) and incubation took place in a Cytomat2 C‐LiN ToS automated incubator (Thermo Scientific, Langenselbold, Germany) at 30°C and 800 rpm.

### 2.2 Cloning

MoClo reactions were performed as previously described (Iverson et al. 2016) with 10 fmol per part and in 10 μL total reaction volume. All level 0 parts were cloned in the appropriate CIDAR MoClo DVA vectors via Gibson assembly or BbsI‐MoClo. BsaI restriction sites present in the different parts were eliminated via PCR and site‐directed mutagenesis while preserving the original amino acid sequence of the open reading frames. The fusion sites are the same as described in Iverson et al. 2016: A (5′‐GGAG‐3′), B (5′‐TACT‐3′), C (5′‐AATG‐3′), D (5′‐AGGT‐3′), E (5′‐GCTT‐3′), and F (5′‐CGCT‐3′) (Iverson et al. 2016). The level 1 Golden Gate‐based shuttle expression plasmids used for MoClo (pTUL and pTUC vectors) were derived from the pPBEx2 vector (Bakkes et al. 2020), which can be propagated in *E. coli* and *C. glutamicum* . The P*lac*‐*lacZ*' region or the P*syn*‐*mCherry* were cloned via Gibson assembly into the PCR amplified pPBEx2 vector and contained the appropriate surrounding MoClo fusion sites (A–E, A–F, C–E, or C–F). pTUL_CE_ and pTUC_CE_ vectors containing fusion sites C and E allowed for the assembly of two parts (SP and gene of interest) and already possessed a promoter (T7) and an RBS as well as the split GFP_11_ tag for protein quantification. Vectors containing fusion sites A and E and A and F allowed the assembly of four or five parts, respectively, specifically a promoter, RBS, SP, gene of interest, and a C‐terminal tag. All vectors contained two transcriptional terminators, one for the native RNA polymerase and an additional one for the T7 RNA polymerase.

### 2.3 Enzymatic Assays

The cutinase assay was performed as previously described (Caspers et al. 2010; Bakkes et al. 2020). Briefly, supernatants were 30–50‐fold diluted in Sørensen's phosphate buffer pH 8.0. The substrate, *p*‐nitrophenyl palmitate (*p*NPP), was first dissolved in isopropanol and then diluted in Sørensen's phosphate buffer pH 8.0 containing 1.1 mg m L^−1^ gum arabic and 2.3 mg m L^−1^ sodium deoxycholate to a final concentration of 0.8 mM. Next, 20 μL of diluted supernatant was quickly mixed with 180 μL of substrate solution in a 96‐well plate and incubated at 37°C. The increase in absorbance at 410 nm was recorded on a Tecan Infinite M1000 Pro microplate reader (Tecan Group Ltd., Männedorf, Switzerland). For the *p*‐nitrophenyl butyrate (*p*NPB) assay used for determining the PETase activity, the supernatants were diluted 10‐fold in Sørensen's phosphate buffer pH 7.5 and 0.5 mM *p*NPB was used as substrate. 20 μL of diluted supernatant was quickly mixed with 180 μL of substrate solution in a 96‐well plate and incubated at 25°C. The increase in absorbance at 405 nm was recorded on a Tecan Infinite M1000 Pro microplate reader. In both cases the enzymatic activity was calculated from the slope of the linear increase region of the enzymatic assay using a molar extinction coefficient of 15,000 M^−1^ cm^−1^ (Caspers et al. 2010; Bakkes et al. 2020).

### 2.4 Split GFP Assay

Production of the detector solution (GFP_1‐10_) as well as the split GFP assay was performed as described previously (Knapp et al. 2017). Briefly, 20 μL of clarified supernatant were mixed with 180 μL of GFP_1‐10_ detector solution in a black flat‐bottom 96‐well microtiter plate covered with a gas permeable membrane and incubated for 24 h at room 20°C. Fluorescence was measured using either a Tecan Infinite M1000 Pro or an Agilent BioTek Synergy H1 (Agilent Technologies Inc., Ratingen, Germany) with an excitation wavelength of 485 nm and emission wavelength of 510 nm. Whenever stated, the split GFP fluorescence values were calculated relative to those of the NprE‐cutinase‐GFP_11_ control cultivated in the exact same conditions and present in the same 96‐well plate.

### 2.5 SDS‐PAGE of Precipitated Supernatants

Protein present in the culture supernatants (300 μL) was precipitated with 10% (v/v) trichloroacetic acid (30 μL) at 4°C for a minimum of 1 h. Samples were then centrifuged at 20,000 *g* at 4°C for 30 min. The pellet was washed with 300 μL acetone, centrifuged at 20,000 *g* at 4°C for 15 min, dried, and resuspended in 10 mM Tris–HCl pH 7.5 and 1× Laemmli sample buffer (Laemmli 1970). The protein present in the supernatant equivalent to 0.5 mL culture of OD_600_ 1 mL^−1^, as measured at the end of cultivation and before centrifugation, was loaded for each sample on a 12.5% SDS‐PAGE after 5 min incubation at 95°C. Proteins were detected by Coomassie Brilliant Blue staining.

### 2.6 Cutinase, PHL7 and Hoce^F265A^ Purification

Strains of *C. glutamicum* MB001(DE3) containing either pTUC_CF_‐SP025‐cutinase‐TEV‐FL‐10×His, or pTUC_CF_‐SP030‐PHL7‐TEV‐FL‐10×His, or pTUC_CF_‐SP025‐Hoce^F265A^‐TEV‐FL‐10×His were cultivated following the same steps as described above (see 4.1) in a total volume of 50 mL. After the cultivation, the supernatants were collected via centrifugation for 5 min at 3000 *g* and filtered via a membrane filter with 0.2 μm pore size. After adjusting the imidazole concentration of the clarified supernatants to 20 mM, these were injected on a 1 mL HisTrap HP (Cytiva, Marlborough, MA, USA) column at 1 mL min^−1^ via an Äkta Pure 25 chromatography system (Cytiva, Marlborough, MA, USA). The column was subsequently washed with 20 column volumes of wash buffer (50 mM NaH_2_PO_4_, 500 mM NaCl, 20 mM imidazole, pH 8.0), and the protein eluted in one step with elution buffer (50 mM NaH_2_PO_4_, 500 mM NaCl, 500 mM imidazole, pH 8.0). Protein concentration was estimated via the Unicorn software 7.11 (Build 7.11.0.1097) of the Äkta Pure 25 chromatography system, taking into account the extinction coefficient of each protein calculated via the ProtParam tool (Gasteiger et al. 2005): cutinase‐TEV‐FL‐10×His (*ε*_280nm_ = 15.93 mM^−1^ cm^−1^), PHL7‐TEV‐FL‐10×His (*ε*_280nm_ = 35.41 mM^−1^ cm^−1^), and Hoce^F265A^‐TEV‐FL‐10×His (*ε*_280nm_ = 48.36 mM^−1^ cm^−1^).

## 3 Results and Discussion

### 3.1 Modular *C. glutamicum* Sec‐Dependent Signal Peptide Library

**FIGURE 1:**
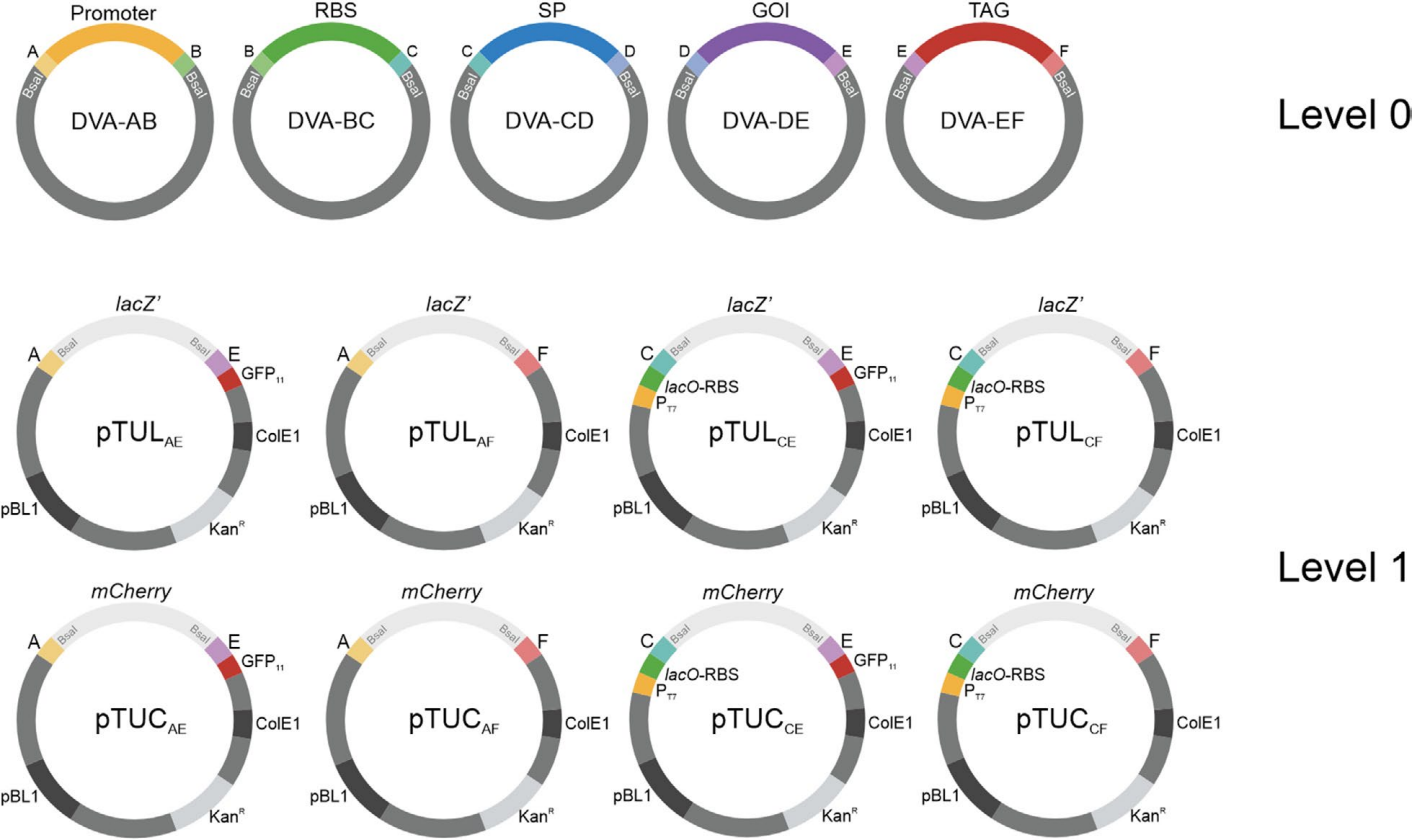
Modular cloning system for use in *C. glutamicum* . Promoters and ribosome binding sites (RBS) commonly used in *C. glutamicum* or *E. coli* , 30 *C. glutamicum* SPs, genes of interest (GOI) and purification tags (TAG) were cloned into the CIDAR level 0 vectors (DVAs (Iverson et al. 2016)). A series of transcriptional unit vectors (pTU) suitable for replication in both *E. coli* and *C. glutamicum* (ColE1 and pBL1 origin of replication) which carry different fusion sites (AE, AF, CE and CF), the kanamycin resistance gene (Kan^R^), the *lacI* repressor gene, and a reporter gene (*lacZ*' in pTUL vectors or *mCherry* in pTUC vectors under the control of the *lac* or *syn* (Henke et al. 2021) promoters, respectively, and cloned in the opposite orientation to the promoter and the GOI in the final construct) were constructed as level 1 vectors.

To create a modular *C. glutamicum* SP library we built upon the CIDAR MoClo kit for *E. coli* (Iverson et al. 2016) since this system was previously established at the AutoBioTech platform for automated strain engineering (Rosch et al. 2024). For this purpose, a number of compatible parts (promoters, ribosome binding sites, SPs, tags) as well as single transcriptional unit vectors suitable for use in *C. glutamicum* and *E. coli* were generated (Figure 1). All level 0 parts were cloned into the respective CIDAR MoClo destination vector‐ampicillin (DVA) and therefore each of the different parts can be assembled in a directional manner and possess the same surrounding fusion sites as in the CIDAR MoClo system (Iverson et al. 2016). This allowed us to have a system with promoters (DVA‐AB), RBSs (DVA‐BC), SPs or N‐terminal tags (DVA‐CD), genes of interest (DVA‐DE) and C‐terminal protein tags (DVA‐EF) as modules (Figure 1).

In addition, we created a dedicated level 1 shuttle ( *E. coli* / *C. glutamicum* ) transcriptional unit vector (pTUL_CE_, Figure 1) that allowed the assembly of two parts, a SP and a gene of interest, via Type IIS BsaI‐dependent Golden Gate modular cloning. This specific SP library‐screening vector (pTUL_CE_) is derived from the pPBEx2 vector (Bakkes et al. 2020). It includes a T7‐RNA polymerase‐dependent promoter, the *lac* operator regulatory region and an RBS, the *lacZ*' for α‐complementation surrounded by the fusion sites C and E, as well as a flexible linker and a GFP_11_ detection tag. The GFP_11_ tag when translationally fused to the C‐terminus of the target protein allows easy quantification of the amount of secreted protein present in the supernatant via the split GFP assay (Cabantous et al. 2005; Knapp et al. 2017).

*C. glutamicum* SP sequences were identified by analysing all annotated protein sequences of ATCC 13032 through the SignalP 6.0 algorithm (Teufel et al. 2022). SignalP 6.0 identified 222 proteins containing potential SPs (Table S1). Of these 222, 111 were predicted to be “standard” secretory SPs transported by the Sec translocon and cleaved by signal peptidase I; 96 lipoprotein SPs transported by the Sec translocon and cleaved by signal peptidase II; 5 Tat SPs transported by the Tat translocon and cleaved by signal peptidase I; 10 Tat lipoprotein SPs transported by the Tat translocon and cleaved by signal peptidase II; and no pilin and pilin‐like SPs transported by the Sec translocon and cleaved by Signal Peptidase III. The top 30 Sec‐dependent SPs (SP001–SP031) were cloned into the CIDAR level 0 DVA‐CD vector (Iverson et al. 2016) to generate a partial *C. glutamicum* SP library (Figure 1, Table 1). SP003 and SP005 have identical amino acid sequences and therefore only SP003 was tested (Table 1).

**TABLE 1:** List of signal peptides used in this study^a^.

Designation	Gene ID	Probability	Signal peptide amino acid sequence
SP001	Cg0765	0.999171	MRFSRVLPALLITTAVSIPTASA
SP002	Cg2069	0.999138	MRLVRRLVGVSAVMLLAIGVASPVAQA
SP003	Cg1942	0.999134	MRKLASIGIAIALSLAITPTIQA
SP004	Cg1247	0.999119	VTKTLPRLLTVAAALAIALPATPVASA
SP005	Cg1517	0.999116	MRKLASIGIAIALSLAITPTIQA
SP006	Cg0782	0.999115	MRRSLRHGFTALLTTWALLLPTVAVA
SP007	Cg2992	0.999109	MSSRNYRSIGFILLFLAVLCLFAAVFA
SP008	Cg1108	0.999102	MKKLRFATIAAATVALTASLTPSASA
SP009	Cg0175	0.999099	MRNQTIAAVAALVLLTAATPAIA
SP010	Cg0311	0.999079	MRMKSIAAIAIATAALAGGTGVASA
SP011	Cg0726	0.999070	MRISSKLVTTALLAAISLFGISTAQA
SP012	Cg0107	0.999066	MSFLNSAKTKTVALTATFVGAATLATPAIASA
SP013	Cg2336	0.999064	LRRSTLTLVTASAVALSVFTPVAQA
SP014	Cg0793	0.999046	MKKAMRAAIGLAVSTAMTFGIAPSAHA
SP015	Cg1936	0.999034	MSLKTRRIFGALAVSLSISFSAIATPAASA
SP016	Cg2223	0.999023	MLNFMPFRLMWILALTFTLTLLTSPALA
SP017	Cg0905	0.999022	MKLFSQAAGIIAAALLVTGGIAPVAQG
SP018	Cg1347	0.999021	MRPSSRPLGLVLCTALASTIITVPAASA
SP019	Cg0161	0.999009	VKIKSVFLSTALSASLLLGITPPVLG
SP020	Cg2061	0.999003	MKLFSKAAGVIAAALLVAGGIAPVAQG
SP021	Cg0413	0.999001	MKLLRRIAAPAIALGIAMSTIVTPSTAGA
SP022	Cg0470	0.998995	MNKLATRALVALTGSAIAMTGLTVVSANA
SP023	Cg3057	0.998977	MKLSKATRCLVALMFAAPLMSAPLANA
SP024	Cg0650	0.998973	MAKNSRIRYSASIKRAAAAILTAAATSVALIAVPATASA
SP025	Cg1911	0.998951	VLTSIRASTTVIALSVLISTLTFASPSEA
SP026	Cg2959	0.998948	LKSKKLLSVLTAVALSGGVVTTTAIVSPSIVSA
SP027	Cg2052	0.998943	MLRKTVTGGIVALIATATLMNSVSSA
SP028	Cg0665	0.998940	MRRLIAVSLAALFMLASTPATRA
SP029	Cg1109	0.998935	MKLSHRIAAMAATAGITVAAFAAPASA
SP030	Cg0411	0.998928	MRKTLITMLATTAIAFSAISPVQA
SP031	Cg1159	0.998917	MSQPLSKRLSIRKALASAFIVALAFSLSPVAKAQAN
NprE	NprE ( *B. subtilis* )	0.998929	MGLGKKLSVAVAASFMSLSISLPGVQA

^a^ The table shows the top SPs, which are predicted to direct protein export via the Sec translocon and be cleaved via signal peptidase I, identified for *C. glutamicum* and the NprE SP from *B. subtilis* . The probability and type of SP were calculated with the SignalP 6.0 algorithm (Teufel et al. 2022). Note that SP003 and SP005 have identical amino acids.

**FIGURE 2:**
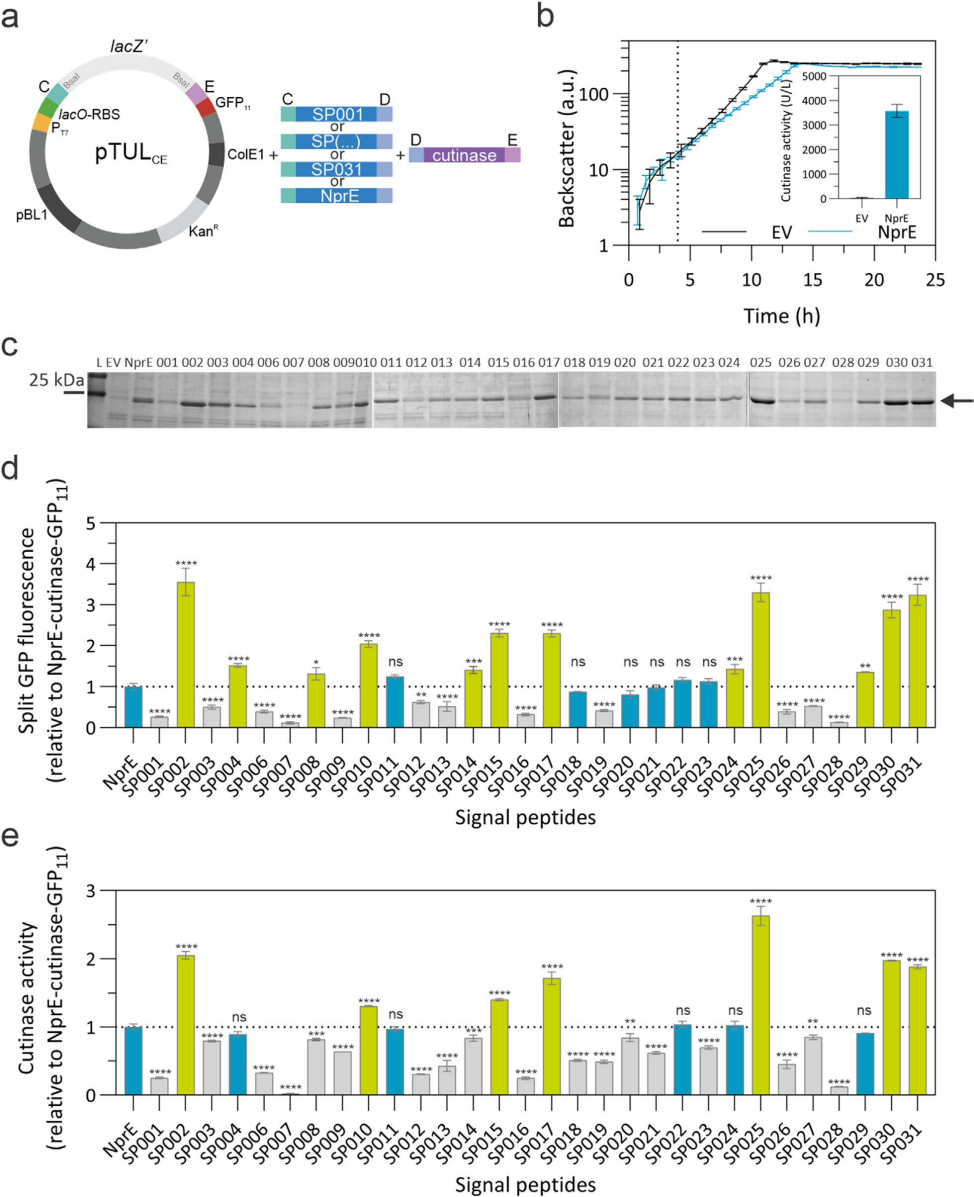
Evaluation of the modular *C. glutamicum* SP library. (a) Schematics of the MoClo assembly for the constructs used in the experiments shown in this figure. (b) Growth (backscatter) of strains MB001(DE3) carrying either an empty vector (EV, pTUL_CE_) control or the pTUL_CE_‐NprE‐cutinase‐GFP_11_ construct in CGXII medium +20 g/L glucose (w/v) in a BioLector for 24 h. Protein production was induced with 250 μM IPTG at 4 h (dotted line). At the end of the cultivation the supernatants were collected and analysed for cutinase activity (b, inset) and by SDS‐PAGE after protein precipitation (c). (d) Split GFP assay of the supernatants collected from strain MB001(DE3) carrying each of the constructs containing the SP indicated in the x‐axis. Cultures were grown in the same conditions as in b. All results are plotted relative to the split GFP activity of the control construct pTUL_CE_‐NprE‐cutinase‐GFP_11_ (dotted line). (e) Cutinase activity assay of the same supernatants used in d. All results are plotted relative to the cutinase activity of the control construct NprE‐cutinase‐GFP_11_ (dotted line). For all experiments the mean and standard deviation of three biological replicates are shown. In d and e the data was analysed in GraphPad Prism 10.3.1, a one‐way ANOVA (Dunnett) test was used to compare each of the SPs to NprE. SPs that did not significantly (ns) differ from NprE are indicated in blue. All the others were coloured depending on whether they showed decreased (grey) or increased (green) amount of protein production or cutinase activity. Adjusted *p* < 0.0001 (****), < 0.001 (***), < 0.01 (**), < 0.1 (*) is indicated above each bar.

The library was initially tested with the cutinase enzyme from *Fusarium solani pisi* since it has been extensively used as a model enzyme to evaluate the efficiency of protein secretion in *C. glutamicum* (Hemmerich et al. 2016, 2019; Bakkes et al. 2021). The neutral protease (NprE) SP from *B. subtilis* was included as a positive control as it has been shown to be one of the best SPs for the secretion of the cutinase enzyme by *C. glutamicum* (Hemmerich et al. 2019). For the library screening, all level 1 vectors were assembled via MoClo by combining pTUL_CE_ with one of the DVA‐CD vectors encoding one of the selected SPs and the DVA‐DE vector carrying the cutinase gene as GOI (Figure 2a). The resulting plasmids were isolated from *E. coli* and used to transform the genome‐reduced *C. glutamicum* MB001(DE3) strain which is suitable for T7‐dependent gene expression (Kortmann et al. 2015). We started by testing the efficiency of the NprE SP to drive cutinase‐GFP_11_ secretion. For this purpose, *C. glutamicum* MB001(DE3) carrying either a vector control (pTUL_CE_) or a pTUL_CE_ derivative encoding NprE‐cutinase‐GFP_11_ was cultivated in CGXII minimal medium supplemented with glucose and protein production induced via IPTG addition (250 μM). Overexpression of the fusion construct resulted in slightly slower growth when compared to the empty vector‐containing strain (Figure 2b). Cutinase secretion was examined via activity assay and SDS‐PAGE of culture supernatants. As observed in Figure 2b—inset and Figure 2c, extracellular cutinase activity of ∼3200 U/L and an overproduced protein with the expected size of ∼25 kDa was detected in the supernatant of the NprE‐cutinase‐GFP_11_ cultures, but not in the control cultures (EV). The cutinase activity obtained is similar to what has been previously reported in the literature when using *C. glutamicum* and the SP NprE to drive cutinase secretion (Bakkes et al. 2021; Müller et al. 2022).

The 30 *C. glutamicum* SPs that were selected based on their SignalP 6.0 score were used to construct 30 vectors encoding each one of the SPs present in the library translationally fused to the cutinase and the GFP_11_ tag and tested under the same conditions as NprE. For an easier comparison across experiments, all results obtained for the *C. glutamicum* SPs are shown as relative to those obtained for NprE, which was included as control in all experiments. Most strains carrying the different SP‐cutinase‐GFP_11_ constructs grew similarly to the empty vector control, with just a few showing a more pronounced growth defect upon induction of protein production, such as those encoding the SP003, SP007, SP009, SP011, SP016, and SP020 protein fusions (Figure S1). The 30 SPs yielded variable levels of cutinase secretion and activity as examined by split GFP and activity assays, respectively (Figure 2d,e). In general, there was a very good correlation between the secreted protein amount as examined by SDS‐PAGE (Figure 2c), the split GFP assay (Figure 2d), and the cutinase activity (Figure 2e). SP001, SP007, SP016, and SP028 were among the worst performing SPs both in terms of amount of protein secreted as in cutinase activity. Among the tested variants, SPs SP002, SP025, SP030, and SP031 showed the highest efficiency in secreting cutinase‐GFP_11_, resulting in 2.9 to 3.3‐fold increases in protein yield (Figure 2d) and 1.9 to 2.8‐fold increases in cutinase activity (Figure 2e) in the supernatant, compared to NprE. The fact that we could identify several *C. glutamicum* native SPs that outperformed the *B. subtilis* NprE SP clearly suggests that it is worth developing organism‐specific SP libraries.

### 3.2 Screening of the Best Promoter and RBS for Cutinase Production

Protein production and its secretion are two major stress factors in recombinant protein production. On the one hand, protein overproduction can lead to significant growth impairment with accumulation of inactive or misfolded proteins that are often directed for degradation. On the other hand, overproduction of exported proteins can lead to excessive secretion‐associated stress with detrimental consequences for growth and also for the overall yield of protein produced and secreted. Optimization of production and secretion stress level can lead to an improvement of up to 70% in the level of secreted protein (Sosa‐Carrillo et al. 2023). Therefore controlling the amount of protein produced is mandatory for maximisation of secreted recombinant protein production. In this context, screening different promoters and RBSs can be useful in determining the best expression level. In this study, we evaluated several promoters and RBSs from the CIDAR Addgene kit (#1000000059), originally developed for *E. coli* (Anderson promoter collection [iGEM]: J23100 [strong], J23102 [strong], J23106 [medium], J23116 [medium‐weak], and J23103 [weak]; RBS collection [iGEM]: B0032 [medium] and B0033 [weak]; bicistronic design elements: BCD2 [strong], BCD8 [weak], BCD12 [medium] (Mutalik et al. 2013)) along with three promoters commonly used for gene expression and protein production in *C. glutamicum* (P_T7_, P_*tac*_ and P_*syn*_ (Henke et al. 2021)) and the consensus RBS (AGGAG) for *C. glutamicum* (Pfeifer‐Sancar et al. 2013) carrying (*lacO*‐RBS_C.g_.) or not carrying the *lacO* (RBS_C.g_.) regulatory region. Bicistronic design elements are used to enhance recombinant protein production by using an RBS and a short peptide sequence upstream of a second RBS and the target gene since the likelihood of mRNA secondary structures that impair ribosome binding at the second RBS is lower and therefore allows for higher and more consistent translation efficiency (Mutalik et al. 2013). All parts (promoter, RBS, SP, GOI, vector) were assembled via MoClo, using SP025 as SP. We used a modified T7 promoter that carried an optimised sequence downstream of the transcriptional start site to promote maximal activity (Conrad et al. 2020) (Table S2).

**FIGURE 3:**
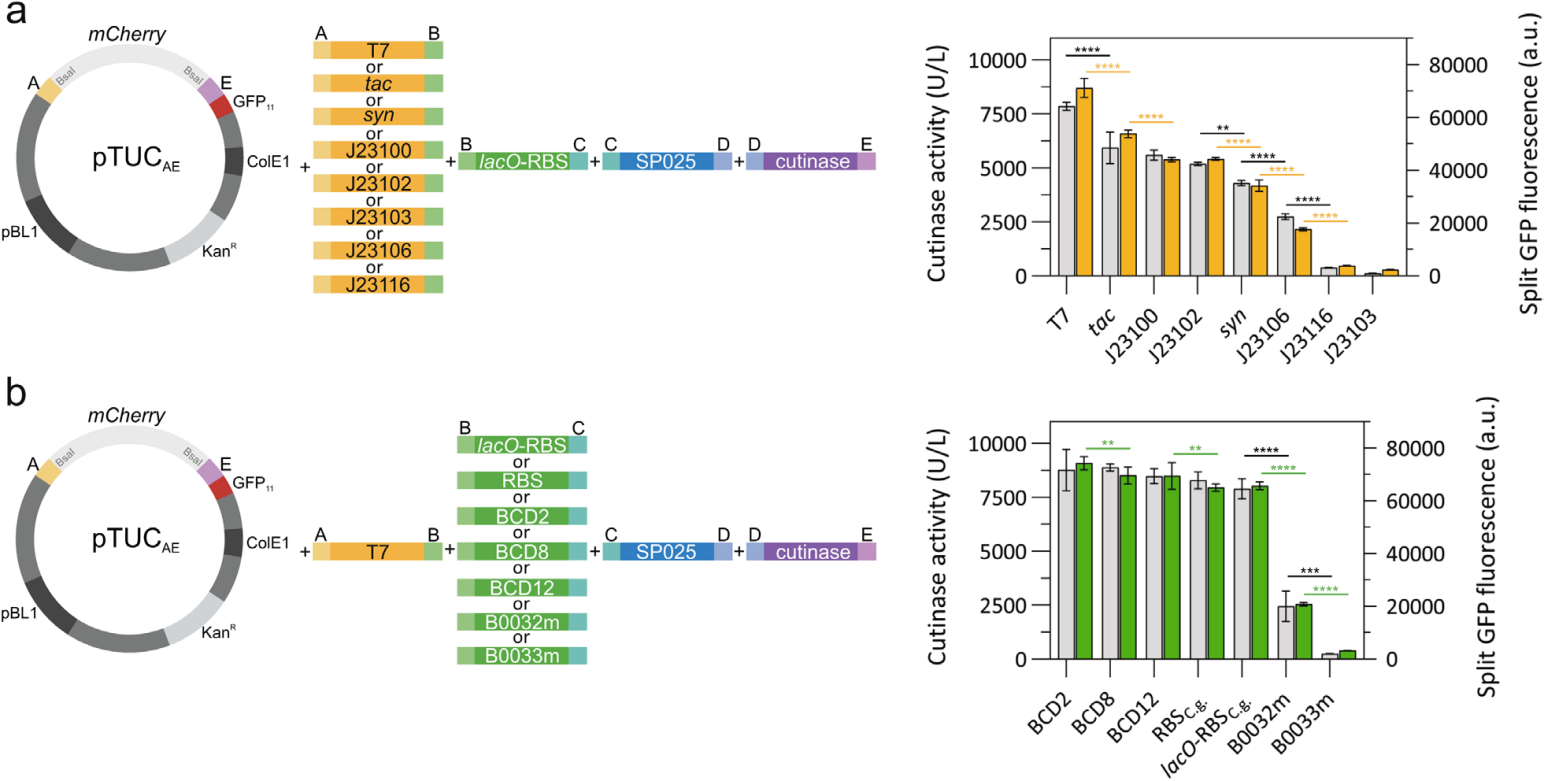
Evaluation of different promoters and RBSs for cutinase secretion in *C. glutamicum* . Three clones from each construct were cultivated in CGXII medium +20 g/L glucose (w/v) and protein production was induced with 250 μM IPTG at 4 h. At the end of the cultivation after 24 h the supernatants were collected and analysed for cutinase activity and by the split GFP assay. (a) Graphical representation of the different parts used in the assemblies for the promoters test (left). Cutinase activity assay (grey) and split GFP assay (yellow) of each different promoter construct (right). Each of the promoters tested is indicated in the x‐axis. (b) Graphical representation of the different parts used in the assemblies for the RBS test (left). Cutinase activity assay (grey) and split GFP assay (green) of each of the different RBSs tested (right). Each of the RBS tested is indicated in the x‐axis. Shown are mean values and standard deviation of at least three replicates. The data was analysed in GraphPad Prism 10.3.1, a one‐way ANOVA (Šídák) test was used to compare the datasets. Adjusted *p* < 0.0001 (****), < 0.001 (***), < 0.01 (**) are indicated above the bars, for all other comparisons differences were not significant and are not indicated on the graph.

As shown in Figure 3a, P_T7_ was the promoter that resulted in the highest cutinase activity and split GFP signal detected in the culture supernatant. All *E. coli* promoters tested were active in *C. glutamicum* and showed the same trend as in *E. coli* regarding their classification as strong, medium, or weak (Figure 3a). The same cannot be said for the bicistronic design constructs (BCD2, BCD12, and BCD8), where regardless of their previous classification for *E. coli* as strong, medium, and weak, they all showed the same strength in *C. glutamicum* (Figure 3b). Disparity between BCD expression in *E. coli* and *C. glutamicum* has previously been observed (Sun et al. 2020), suggesting that there are important differences in translation between the two organisms. Specific *C. glutamicum* BCD could be tested, as those described in (Liu et al. 2017, 2023; Sun et al. 2020). As for the other RBS constructs, either those derived from the *C. glutamicum* consensus sequence with or without the *lacO* regulatory region or those classified as medium (B0032m) and weak (B0033m) for *E. coli* , all followed the expected trend in terms of the amount of cutinase produced and its activity (Figure 3b).

### 3.3 One‐Pot MoClo Assembly for Rapid Identification of the Best SP for Cutinase and Four PETases

To rapidly screen for the best SP and significantly reduce the associated cost of screening, we tested the feasibility of, on one hand, transforming the MoClo reactions straight into *C. glutamicum* , bypassing the necessity for *E. coli* transformation and plasmid isolation. And, on the other hand, instead of performing individual reactions for each SP, establishing a one‐pot reaction with all SPs at once, followed by the screening of an appropriate number of clones to identify the best SP. This approach allowed a significant reduction in time and material used. To address the first point, we used the newly created transcriptional unit vectors that carried *mCherry* instead of *lacZ*α (Figure 1) to directly select the *C. glutamicum* positive clones via pink/white selection. Since overexpression of some GOI can be detrimental to the bacterial growth, mCherry was cloned under the control of the *C. glutamicum* constitutive promoter (P*syn* (Henke et al. 2021)) so that induction was not needed to differentiate between the empty vector and those that contained the desired constructs.

**FIGURE 4:**
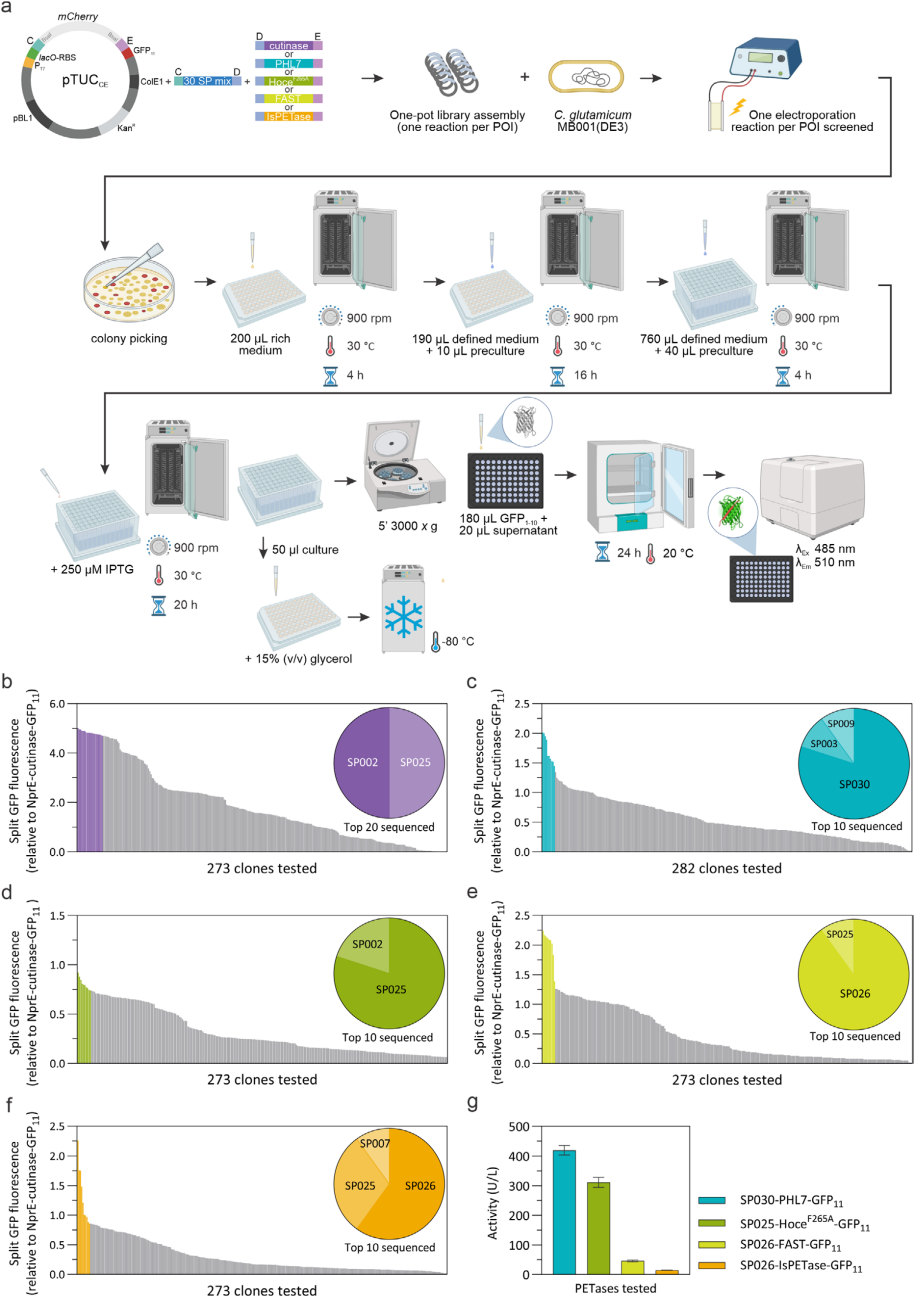
Automated secretion screening for the identification of the best SP for a protein of interest (POI). (a) Schematics of the one‐pot MoClo assembly, *C. glutamicum* transformation and the subsequent automated procedure performed at the AutoBioTech platform (Rosch et al. 2024). A minimum of 273 clones were tested for each POI corresponding to twice the minimum number of clones (*N*) needed to ensure that each SP is represented at least once with a probability of 0.99 according to the Clarke‐Carbon equation (Clarke and Carbon 1976), *N* = ln(1‐*p*)/ln(1‐*f*), where *p* is the desired probability and *f* is the fractional proportion of a SP in the library. Cultivations were performed in either rich medium (BHIS) or defined medium CGXII+ 20 g/L glucose (w/v) both in the presence of 25 μg/mL kanamycin. Secretion of cutinase‐GFP_11_ (b), PHL7‐GFP_11_ (c), Hoce^F265A^‐GFP_11_ (d), FAST‐PETase‐GFP_11_ (e), and IsPETase‐GFP_11_ (f) was detected via split GFP assay. All results are plotted relative to the split GFP fluorescence value of the control construct NprE‐cutinase‐GFP_11_. The top clones sent for sequencing are shown in colour whereas all other clones are shown in grey. The sequencing results are represented in the inset as a pie chart in which each colour represents the proportion of each identified SP. (g) *p*NPB activity assay performed with the supernatant of the top candidate for PHL7‐GFP_11_, Hoce^F265A^‐GFP_11_, FAST‐PETase‐GFP_11_, and IsPETase‐GFP_11_. Cutinase‐GFP_11_ activity for constructs with SP002 and SP025 is shown in Figure 2e. The mean and standard deviation of three biological replicates are shown.

For this MoClo reaction with all 30 SPs, it was crucial to ensure that all vectors carrying the SP‐encoding DNA fragments were present at equimolar amounts to have an equal probability of assembly into the final construct. *C. glutamicum* MB001(DE3) was then directly transformed with this MoClo reaction, and 273 or 282 random clones for each enzyme tested were picked and screened on the AutoBioTech platform through the development and implementation of custom automated routines covering colony picking, pre‐culture inoculation, main culture cultivation and induction, supernatant isolation, and split GFP assay incubation and detection (Figure 4a). This approach was initially tested for the secretion of cutinase. The empty vector pTUC_CE_ was included as a negative control and, as previously described, the split GFP value of cutinase‐GFP_11_ secreted using NprE as SP was used to normalise all results. Sequencing of the top 20 clones showed that two SPs were present, SP002 and SP025, with equal prevalence (Figure 4b). Interestingly, SP002 and SP025 were also the two SPs that led to the highest protein abundance (Figure 2d) and cutinase activity (Figure 2e) when tested individually. This result shows that the one‐pot random approach is an efficient way to identify the best SP for the secretion of a protein of interest.

The same procedure was used to test secretion of additional PETase enzymes, PHL7 (Sonnendecker et al. 2022), Hoce^F265A^ (Molitor 2024) as well as the *Ideonella sakaiensis* PETase (Yoshida et al. 2016) and its improved variant, the FAST‐PETase (Lu et al. 2022). In addition, the number of clones sent for sequencing was reduced to only the top ten since it was deemed sufficient for the identification of the best SP. Three SPs were in the top ten for PHL7‐GFP_11_ secretion (Figure 4c), SP003 (*n* = 1), SP009 (*n* = 1) and the most often identified, SP030 (*n* = 8). Just like for the cutinase‐GFP_11_, SP002 (*n* = 2) and SP025 (*n* = 8) were identified as the best SPs for Hoce^F265A^‐GFP_11_ secretion (Figure 4d). For the FAST‐PETase‐GFP_11_ and for the IsPETase‐GFP_11_, since they are so similar, SP026 was identified in both cases (*n* = 9 and *n* = 6, respectively) as the best SP for the secretion of these two enzymes (Figure 4e,f). Presence of the desired POI in the supernatant was examined via SDS‐PAGE of the precipitated supernatants for the top candidates for each POI (Figure S2).

In comparison to the experiments illustrated in Figure 2, the library screenings using the one‐pot MoClo assembly approach were about 4‐fold faster since we bypassed the use of *E. coli* for all cloning steps; only one transformation per library screening was needed, the identity of each construct was determined only for the top performers, and finally, two library screenings could be performed in parallel in our automated setup.

To confirm the presence of active secreted PETase enzyme, each of the supernatants from the cultures with the highest values in the split GFP assay was tested on *p*‐nitrophenyl butyrate as substrate. Activity was detected for all constructs, with PHL7‐GFP_11_ and Hoce^F265A^‐GFP_11_ showing the highest activity (Figure 4g).

**FIGURE 5:**
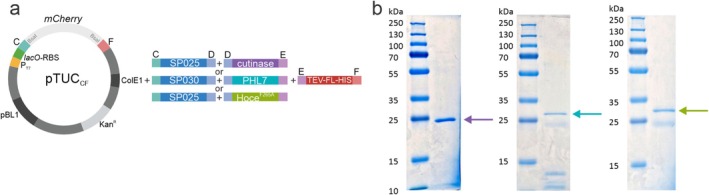
Purification of selected POIs directly from the supernatant of *C. glutamicum* . (a) Schematics of the MoClo assembly. Each of the different POIs was cloned with the respective SP identified in the previous experiments for maximal secretion and an affinity tag into the transcriptional unit vector pTUC_CF_. (b) Coomassie stained SDS‐PAGE of each of the purified enzymes, cutinase‐TEV‐FL‐10×His (~25.0 kDa), PHL7‐TEV‐FL‐10×His (~31.6 kDa), and Hoce^F265A^‐TEV‐FL‐10×His (~32.7 kDa) and the prestained PageRuler Plus protein ladder (Thermo Fisher Scientific Inc., Waltham, US).

Finally, to determine how much protein was produced we assembled constructs carrying the best SP identified for each of three enzymes that showed significant enzymatic activity in the supernatant (cutinase, PHL7 and Hoce^F265A^) and a C‐terminal fragment encoding the Tobacco Echt Virus (TEV) cleavage sequence and a 10× histidine tag separated by a flexible linker (TEV‐FL‐10×His) (Figure 5a). Each protein was secreted and purified directly from the culture supernatant via affinity chromatography. The amount of protein obtained differed, with the cutinase yielding the highest amount of purified protein (72 mg/L) followed by PHL7 (16 mg/L) and then the Hoce‐PE^F265A^ (9 mg/L). All three proteins showed very clean profiles with a few lower molecular weight contaminating bands detected in the purified fractions of PHL7 and Hoce^F265A^ (Figure 5b).

## 4 Conclusion and Outlook

We have developed new synthetic biology tools for *C. glutamicum* that can be used to design and test optimal expression constructs for the production, secretion, and purification of recombinant proteins. Through the development of an organism‐specific SP library and suitable transcriptional unit vectors for parallel screening of multiple modular cloning parts, we could identify superior SPs for the secretion of cutinase by *C. glutamicum* . The use of a one‐pot modular cloning approach and the automated high‐throughput capability of the AutoBioTech platform helped accelerate the discovery time associated with finding the best construct for the secretion of four additional POIs (PETases). Using this strategy, we improved cutinase secretion by ~3‐fold and reduced the optimal SP identification time by at least 4‐fold. This approach can be used to not only identify improved SPs for protein secretion, but also to screen large libraries of SPs in different organisms, which can expand our understanding of SP function and help improve computational tools for the prediction of an optimised SP for the secretion of a particular protein and in the organism of choice.

## Author Contributions

**Susana Matamouros:** conceptualization, investigation, writing – original draft, methodology, validation, writing – review and editing, visualization, data curation. **Julia Tenhaef:** investigation, methodology, visualization, writing – review and editing. **Astrid Bida:** investigation, methodology. **Stephan Noack:** funding acquisition, supervision, writing – review and editing. **Michael Bott:** conceptualization, writing – original draft, writing – review and editing, funding acquisition, supervision.

## Funding

This study was supported by the German Federal Ministry of Education and Research (BMBF; grants 031B1134A and 031B1342F) as part of the innovation lab “AutoBioTech” within the project “Modellregion, BioRevierPLUS: BiookonomieREVIER Innovationscluster Biotechnologie & Kunststofftechnik” and the LipoBiocat project “Maßgeschneiderte Inhaltsstoffe 2‐2: LipoBiocat 2.2 ‐ Verbundvorhaben: Robuste und selektive lipolytische Biokatalysatoren für industrielle Anwendungen ‐ Teilprojekt F”, respectively.

## Conflicts of Interest

The authors declare no conflicts of interest.

## Supporting information


**Figure S1:** Growth profile of 31 *C. glutamicum* MB001(DE3) strains carrying.
**Figure S2:** SDS‐PAGE of the precipitated supernatants from the top candidates.

**Table S1:** List of signal peptides identified in the proteome of *C. glutamicum* ATCC 13032 by SignalP 6.0.

**Table S2:** DNA sequences of all parts constructed for this study.

## Data Availability

The data that support the findings of this study are available from the corresponding authors upon reasonable request.
