# Supplementary material for: Rapid Identification of Superior Endogenous Signal Peptides for Heterologous Protein Secretion by Corynebacterium glutamicum Through Modular Cloning and Automation
Source: Microb Biotechnol. 2026 Jan 16;19(1):e70299. doi: 10.1111/1751-7915.70299 (PMC12810402; doi:10.1111/1751-7915.70299)
Supplement: Supplementary file 1 — Figure S1: Growth profile of 31 C. glutamicum MB001(DE3) strains carrying. Figure S2: SDS‐PAGE of the precipitated supernatants from the top candidates. [file MBT2-19-e70299-s002.pdf]

Supplementary figures Matamouros et al.

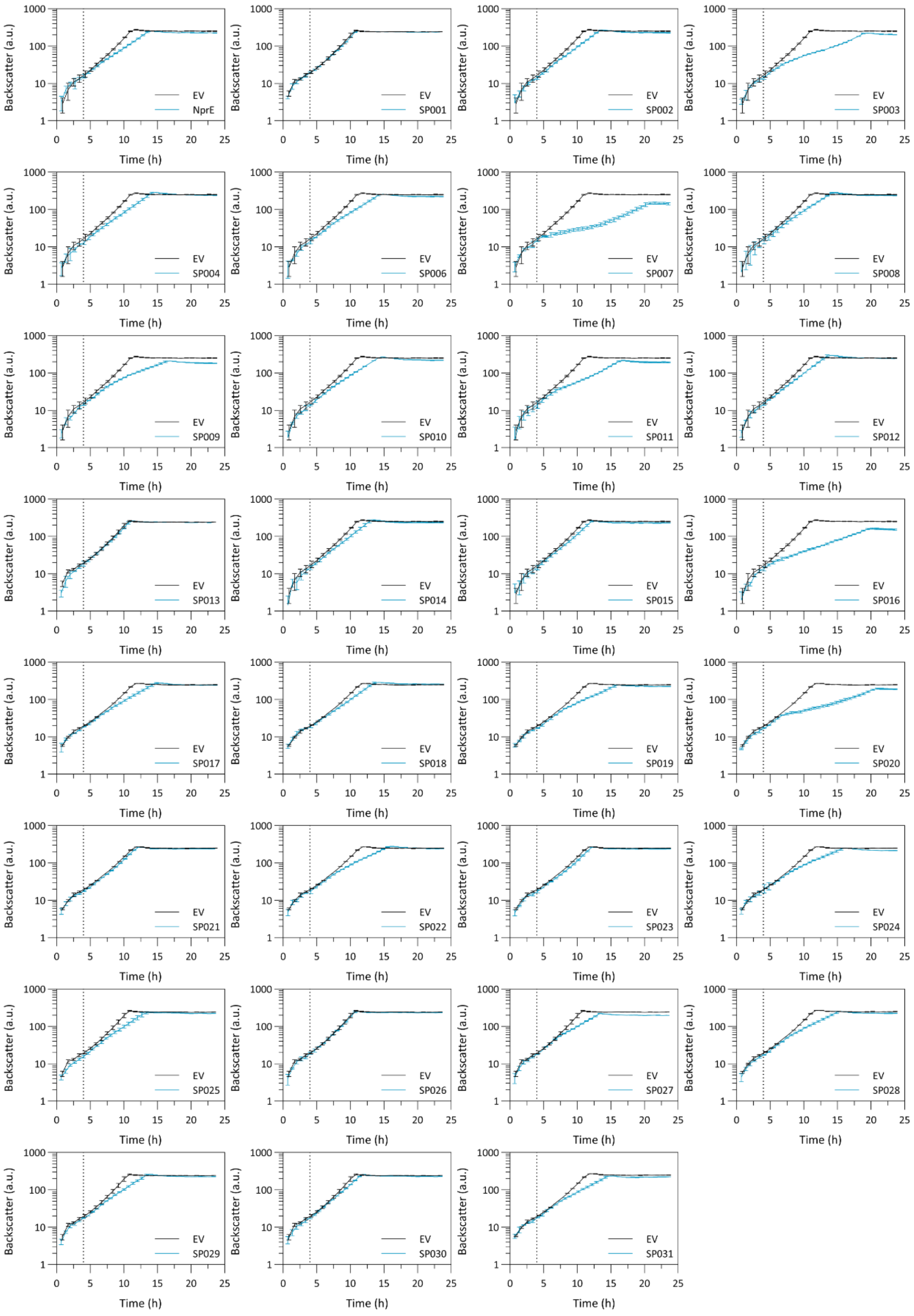

Supplementary Figure 1. Growth profile of 31 *C. glutamicum* MB001(DE3) strains carrying vectors for the NprE-cutinase-GPF_{11} construct and 30 different *C. glutamicum* SP-cutinase-GFP_{11} constructs. Strains were cultivated for 24 h in a BioLector system in CGXII medium + 2% glucose (w/v) + 25 µg/mL kanamycin and induced with 250 µM IPTG at 4 h (dotted line). Results depict the mean and standard deviation of triplicate cultivations.

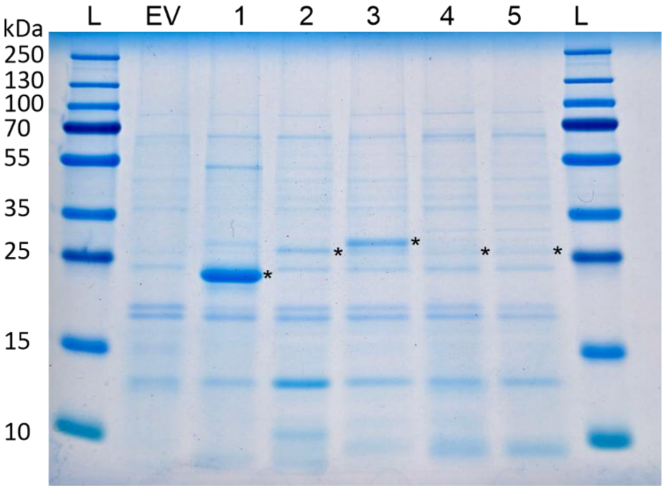

Supplementary Figure 2. SDS-PAGE of the precipitated supernatants from the top candidates of the library screen. *C. glutamicum* MB001(DE3) containing either the empty vector control (EV), SP025-Cutinase-GFP_{11}, 25 kDa (1), SP030-PHL7-GFP_{11}, 31 kDa (2), SP025-Hoce^{F265A}-GFP_{11}, 32 kDa (3), SP026-FAST-PETase-GFP_{11}, 31 kDa (4), and SP026-IsPETase-GFP_{11}, 31 kDa (5) were cultivated as described for the library screen, the supernatants collected, TCA precipitated and run on a 12.5% SDS-PAGE. The prestained PageRuler Plus protein ladder was used as molecular mass standard (L).
